# Evaluation of Multisource Adaptive MRI Fusion for Gross Tumor Volume Delineation of Hepatocellular Carcinoma

**DOI:** 10.3389/fonc.2022.816678

**Published:** 2022-02-25

**Authors:** Andy Lai-Yin Cheung, Lei Zhang, Chenyang Liu, Tian Li, Anson Ho-Yin Cheung, Chun Leung, Angus Kwong-Chuen Leung, Sai-Kit Lam, Victor Ho-Fun Lee, Jing Cai

**Affiliations:** ^1^ Department of Clinical Oncology, Queen Mary Hospital, Hong Kong, Hong Kong SAR, China; ^2^ Department of Health Technology and Informatics, The Hong Kong Polytechnic University, Hong Kong, Hong Kong SAR, China; ^3^ Department of Radiation Oncology, Duke University Medical Center, Durham, NC, United States; ^4^ Medical Physics Graduate Program, Duke University, Durham, NC, United States; ^5^ Medical Physics Graduate Program, Duke Kunshan University, Kunshan, China; ^6^ Radiotherapy and Oncology Centre, Hong Kong Baptist Hospital, Hong Kong, Hong Kong SAR, China; ^7^ AMO Oncology Centre, Hong Kong, Hong Kong SAR, China; ^8^ Department of Clinical Oncology, The University of Hong Kong, Hong Kong, Hong Kong SAR, China

**Keywords:** MRI fusion, tumor contrast, GTV delineation, hepatocellular carcinoma, MR-guided radiotherapy

## Abstract

**Purpose:**

Tumor delineation plays a critical role in radiotherapy for hepatocellular carcinoma (HCC) patients. The incorporation of MRI might improve the ability to correctly identify tumor boundaries and delineation consistency. In this study, we evaluated a novel Multisource Adaptive MRI Fusion (MAMF) method in HCC patients for tumor delineation.

**Methods:**

Ten patients with HCC were included in this study retrospectively. Contrast-enhanced T1-weighted MRI at portal-venous phase (T1W_PP_), contrast-enhanced T1-weighted MRI at 19-min delayed phase (T1W_DP_), T2-weighted (T2W), and diffusion-weighted MRI (DWI) were acquired on a 3T MRI scanner and imported to in-house-developed MAMF software to generate synthetic MR fusion images. The original multi-contrast MR image sets were registered to planning CT by deformable image registration (DIR) using MIM. Four observers independently delineated gross tumor volumes (GTVs) on the planning CT, four original MR image sets, and the fused MRI for all patients. Tumor contrast-to-noise ratio (CNR) and Dice similarity coefficient (DSC) of the GTVs between each observer and a reference observer were measured on the six image sets. Inter-observer and inter-patient mean, SD, and coefficient of variation (CV) of the DSC were evaluated.

**Results:**

Fused MRI showed the highest tumor CNR compared to planning CT and original MR sets in the ten patients. The mean ± SD tumor CNR was 0.72 ± 0.73, 3.66 ± 2.96, 4.13 ± 3.98, 4.10 ± 3.17, 5.25 ± 2.44, and 9.82 ± 4.19 for CT, T1W_PP_, T2W, DWI, T1W_DP_, and fused MRI, respectively. Fused MRI has the minimum inter-observer and inter-patient variations as compared to original MR sets and planning CT sets. GTV delineation inter-observer mean DSC across the ten patients was 0.81 ± 0.09, 0.85 ± 0.08, 0.88 ± 0.04, 0.89 ± 0.08, 0.90 ± 0.04, and 0.95 ± 0.02 for planning CT, T1W_PP_, T2W, DWI, T1W_DP_, and fused MRI, respectively. The patient mean inter-observer CV of DSC was 3.3%, 3.2%, 1.7%, 2.6%, 1.5%, and 0.9% for planning CT, T1W_PP_, T2W, DWI, T1W_DP_, and fused MRI, respectively.

**Conclusion:**

The results demonstrated that the fused MRI generated using the MAMF method can enhance tumor CNR and improve inter-observer consistency of GTV delineation in HCC as compared to planning CT and four commonly used MR image sets (T1W_PP_, T1W_DP_, T2W, and DWI). The MAMF method holds great promise in MRI applications in HCC radiotherapy treatment planning.

## 1 Introduction

Hepatocellular carcinoma (HCC) is the most common primary liver cancer, which is among the most prominent causes of cancer-related deaths worldwide (1). It is one of the deadliest and most aggressive cancer types, with a general 5-year survival of 18%, depending on the stages being diagnosed (2).

Historically, conventional radiotherapy was not the preferred option for the treatment of liver tumors due to the risk of radiation-induced liver damage (RILD) (3). In recent years, a higher radiation dose can be delivered in hypo-fractionated fractions with reduced risk of RILD owing to the adoption of CT, MRI, and image-guided radiotherapy (IGRT) for improved accuracy in target delineation, as well as the use of a rigid immobilizing device for limiting patient movement and cone-beam CT (CBCT)-based image guidance during patient setup (4–11). Highly conformal dose to the target and sparing of the surrounding normal tissues are believed to contribute to the improved outcomes in HCC patients. Target delineation is therefore a critically important step towards precise treatment with high dose conformation, dose escalation, and eventually the success of modern radiotherapy. Indeed, the benefits of dose escalation in both photon and proton therapy for liver malignancies have been demonstrated in multiple clinical trials (12, 13).

In the current clinical practice of liver cancer radiotherapy treatment planning, MRI has been increasingly used alone or in conjunction with CT for tumor and normal tissue delineations because of its superior soft-tissue contrast (9). The contours of the target are firstly created in MR images and then transferred to planning CT images *via* MRI-CT registration. However, MRI might still be prone to inter-sequence and inter-patient variations in image quality and tumor contrast, and potentially inter-observer variations in tumor identification or delineation (14–23).

To overcome these challenges, we have previously developed a Multisource Adaptive MRI Fusion (MAMF) method that is capable of producing a large number of fused MR images with multifaceted image contrasts for RT applications using a limited number of standard MR images as input (21). This method has shown promise in enhancing the image quality of MRI in radiotherapy treatment planning featuring application-specific adaptation and optimization of image contrast (22). In this study, we evaluated the potential clinical efficacy of MAMF in gross tumor volume (GTV) delineation of HCC patients in terms of both tumor contrast optimization and inter-observer variability improvement.

## 2 Methods

### 2.1 Patient Data and Image Acquisition

Ten HCC patients treated with radiotherapy at the Hong Kong Queen Mary Hospital between 2015 and 2019 were retrospectively recruited for this study with Institutional Review Board approval. The distribution of the Child-Pugh score among the enrolled patients was 7 for grade A, 2 for grade B, and 1 for grade C. The CT scan and MRI scans of each patient were performed within 1 week to ensure minimal anatomical changes between scans. During planning CT image acquisition, patients were scanned under a CT scanner (Aquilion/LB, Toshiba, Tokyo, Japan) with a head-first supine position in a vacuum bag with arms raised above their head. The planning MR image acquisition was conducted under a Philips Achieva 3T MRI scanner (Philips Healthcare, Best, The Netherlands). The patient positioning was equivalent to that during planning CT scanning to minimize variations in patient anatomy between CT and MR scans. A series of four MR image sets were acquired including T1-weighted MRI in portal-venous phase (T1W_PP_), T1-weighted MRI in 19-min delay post-contrast (T1W_DP_), T2-weighted MRI (T2W), and diffusion-weighted MRI (DWI).

The details of the imaging protocols for CT and each MR sequence are as follows. Planning CT: tube voltage = 120 kVp; tube current = 50–400 mA; helical scan; field of view (FOV) = 500 mm × 500 mm; slice thickness = 3 mm. T1W_PP_ and T1W_DP_ MRI: pulse sequence = LAVA; 3D mode; time of repetition (TR) = 3.86 ms; time of echo (TE) = 1.79 ms; FOV = 420 mm × 420 mm; slice thickness = 4 mm; flip angle = 12°; bandwidth = 62.5 Hz/pixel. For T1W contrast enhancement, Primovist was deployed as the contrast agent with a concentration of 0.25 mmol/ml and was injected to the patients *via* a rate of 1.5 ml/s. T2W MRI: pulse sequence = FSE-XL; 2D mode; TR = 2,200 ms; TE = 85 ms; FOV = 400 mm × 400 mm; slice thickness = 7 mm; flip angle = 111°; bandwidth = 62.5 Hz/pixel. DWI: pulse sequence: SE; 2D mode; FOV = 400 mm × 400 mm; slice thickness = 7 mm; number of diffusion directions, 3 in 1; b-value = 500 s/mm^2^; NEX = 8.

Respiratory motion management was performed during image acquisitions. The planning CT images were acquired during the end-of-exhalation (EOE) phase of the patient’s respiratory cycle under the breath-holding technique using Varian Real-time Position Management (RPM) (Varian Medical Systems, Palo Alto, CA, USA) in the monitoring of the patient’s breathing motion pattern. T1W_PP_, T1W_DP_, and T2W MR images were acquired during the EOE phase with patient breath-holding. DWI MR images were acquired during the EOE phase using respiratory navigation (Philips Bellows system) due to its longer acquisition time. Prior to both CT and MR image acquisitions, coaching was exercised on patients for assessing breathing stability, breathing consistency, and breath-hold duration, in compliance with an international guideline on stereotactic body radiation therapy (SBRT) from the American Association of Physicists in Medicine (AAPM) Task Group 101 (TG-101) report. The acquired MR images were “stationary” images that represent a single phase of the respiratory cycle of the patients, which were then used for the generation of fused MRI using MAMF (see *Section 2.2*).

### 2.2 Generation of Fused MRI Using Multisource Adaptive MRI Fusion

The MAMF technique consists of five key components: input multiple MRI, image preprocessing, fusion algorithm, adaptation methods, and output fused MRI. For input MRI, the four original MR image sets (T1W_PP_, T1W_DP_, T2W, and DWI) were imported into the in-house-developed MAMF program implemented in Matlab (MathWorks, Natick, MA, USA) to generate a new fused MRI that has enhanced tumor-to-tissue contrast. For image preprocessing, the original four MR image sets were registered to planning CT by deformable image registration (DIR) using MIM Maestro v6.3 (MIM Software Inc., Cleveland, OH, USA). Image intensities were clipped by the 99.5th percentile of each image set and normalized to values between 0 and 1. For image fusion, a linear weighted summation fusion algorithm was used to generate a series of fused MRIs. The fused MRI was synthesized by the following equation:


[1], $$Y_{i}=\Sigma_{k=1}^{K}\;w_{ik}X_{k}$$

where Y is the fused images, X is the input MRI, w ∈ [−1, 1] in an interval of 0.167 is the weight coefficient for each input MRI, and k and i are the indices of input and fused MRI, respectively.

A database of all fused MRI with input image weight coefficients and fused image features was built for each patient. Fused image features in this study included tumor contrast-to-noise ratio (CNR) and liver signal-to-noise ratio (SNR), which are defined as


[2], $$Tumor\;CNR=|\frac{\mu_{Tumor}-\mu_{Liver}}{\sigma_{Liver}}|$$

[3], $$Liver\;SNR=\frac{\mu (Liver)}{\sigma (Liver)}$$

where *μ* and *σ* are the mean and SD of the regional intensities, respectively. Tumor and liver represent the GTV and a nearby homogenous liver region, respectively.

Finally, an output- or feature-driven adaptation approach was used for the fused MRI selection. In this study, for the application of tumor contrast enhancement and GTV delineation, tumor CNR was set to maximum, while liver SNR was set as positive. The optimal image set with the highest tumor CNR and a positive liver SNR in the database was selected for each patient automatically and exported in DICOM format for GTV delineation. The input image weight coefficients were therefore not fixed per imaging techniques or patient. Instead, they were independently optimized to achieve optimal tumor CNR with a positive liver SNR for each patient. More details of the MAMF method could be found in previous publications (21, 22).

### 2.3 Gross Tumor Volume Delineation

Eclipse treatment planning workstation (version 15.6, Varian Medical Systems, Palo Alto, CA, USA) was used for GTV delineation. Four experienced radiation oncologists and medical physicists were recruited from two hospitals to delineate the GTV separately. Identical window and level settings were used for consistency.

### 2.4 Data Analysis

Two main evaluation metrics were used to assess the clinical efficacy of MAMF for GTV delineation: tumor CNR and GTV Dice similarity coefficient (DSC). The tumor CNR was defined in *Section 2.2*. Absolute values of tumor CNR were measured on all six image sets of all patients. Tumor CNR inter-patient (IP) mean, SD, and coefficient of variation (CV) were calculated. The CV of CNR was defined as


[4], $$CNR_{IPCV}=\frac{\sigma (CNR_{i})}{\mu (CNR_{i})}\times 100\%$$

where i = 1 to 10 represents the patient number.

The DSC, defined as the overlap of two volumes divided by their average, was applied to quantify consistencies in the GTV delineation. GTV RT structures of all four observers and all six image sets were exported from the treatment planning system to Python for DSC calculation. The DSC was calculated between the most experienced radiation oncologist contour, which was defined as the reference, and each of three observer contours. The DSC was calculated as follows:


[5], $$DSC_{i}=\frac{2(|GTV_{Ref}|\cap |GTV_{i}|)}{\left|GTV_{Ref}|+|GTV_{i}\right|}$$

where *GTV_Ref_* is the reference GTV and *GTV_i_* is one of the three observer GTVs.

Mean, SD, and CV of the three DSCs were calculated for each image set and each patient. The DSC inter-observer (IO) mean and CV were defined as


[6] $$DSC_{IOmean}=\mu (DSC_{i})$$

[7], $$DSC_{IOCV}=\frac{\sigma (DSC_{i})}{\mu (DSC_{i})}\times 100\%$$

where i = 1 to 3 represents the three observer contours for each image set, and *μ* and *σ* are the mean and SD among the three DSCs. The *DSC_IOmean_* and *DSC_IOCV_* were calculated for all image sets and patients. Paired Student’s t-tests were performed for the *CNR* and *DSC_IOmean_* comparisons between the six image sets.

## 3 Results

### 3.1 Patient Demographic Data

Ten patients were included in the study, including seven male and three female patients. The characteristics of the patients are shown in **Table 1**. Patient age ranged from 58 to 86, and the mean ± SD age was 68.4 ± 9.5 years. Ten HCC tumors were roughly evenly distributed in different liver segments. The GTV volume had a range of 5.9 to 83.0 cm^3^, and the mean ± SD GTV volume was 33.8 ± 27.9 cm^3^.

**Table 1:** Characteristics of HCC patients enrolled in this study.

Characteristic	Finding
Age (year)*	68.4 ± 9.5 (range: 58–86)
Sex	
Male	7
Female	3
GTV volume (cm^3^)*	33.8 ± 27.9 (range: 5.9–83.0)
Tumor location	
Segment 1	0
Segment 2	3
Segment 3	1
Segment 4	2
Segment 5	1
Segment 6	1
Segment 7	2
Segment 8	0

HCC, hepatocellular carcinoma; GTV, gross tumor volume.

*Data: mean ± SD.

### 3.2 Tumor Contrast-to-Noise Ratio

By using the MAMF method, the inter-patient mean ± SD of the optimized weight coefficients for each input imaging technique was as follows: T1W_PP_, 0.63 ± 0.33; T1W_DP_, −0.43 ± 0.21; T2W, 0.50 ± 0.43; and DWI, 0.23 ± 0.38. The details of the weight coefficients for each patient are summarized in **Table 2**.

**Table 2:** Input MRI weight coefficients for the fused MRI with optimal tumor CNR of each patient.

Patient #	T1W_PP_	T1W_DP_	T2W	DWI
1	0.33	−0.33	1.00	0.17
2	1.00	−0.66	0.66	−0.17
3	0.83	−0.66	0.00	1.00
4	1.00	−0.33	0.00	−0.17
5	0.50	−0.17	0.66	0.66
6	1.00	−0.33	−0.17	0.00
7	0.83	−0.33	0.33	0.17
8	0.17	−0.33	0.66	0.17
9	0.33	−0.83	1.00	0.50
10	0.33	−0.33	0.83	0.00
Mean	0.63	−0.43	0.50	0.23
SD	0.33	0.21	0.43	0.38

CNR, contrast-to-noise ratio; T1W_PP_, T1-weighted MRI at portal-venous phase; T1W_DP_, T1-weighted MRI at 19-min delayed phase; T2W, T2-weighted; DWI, diffusion-weighted MRI.

**Figure 1:**
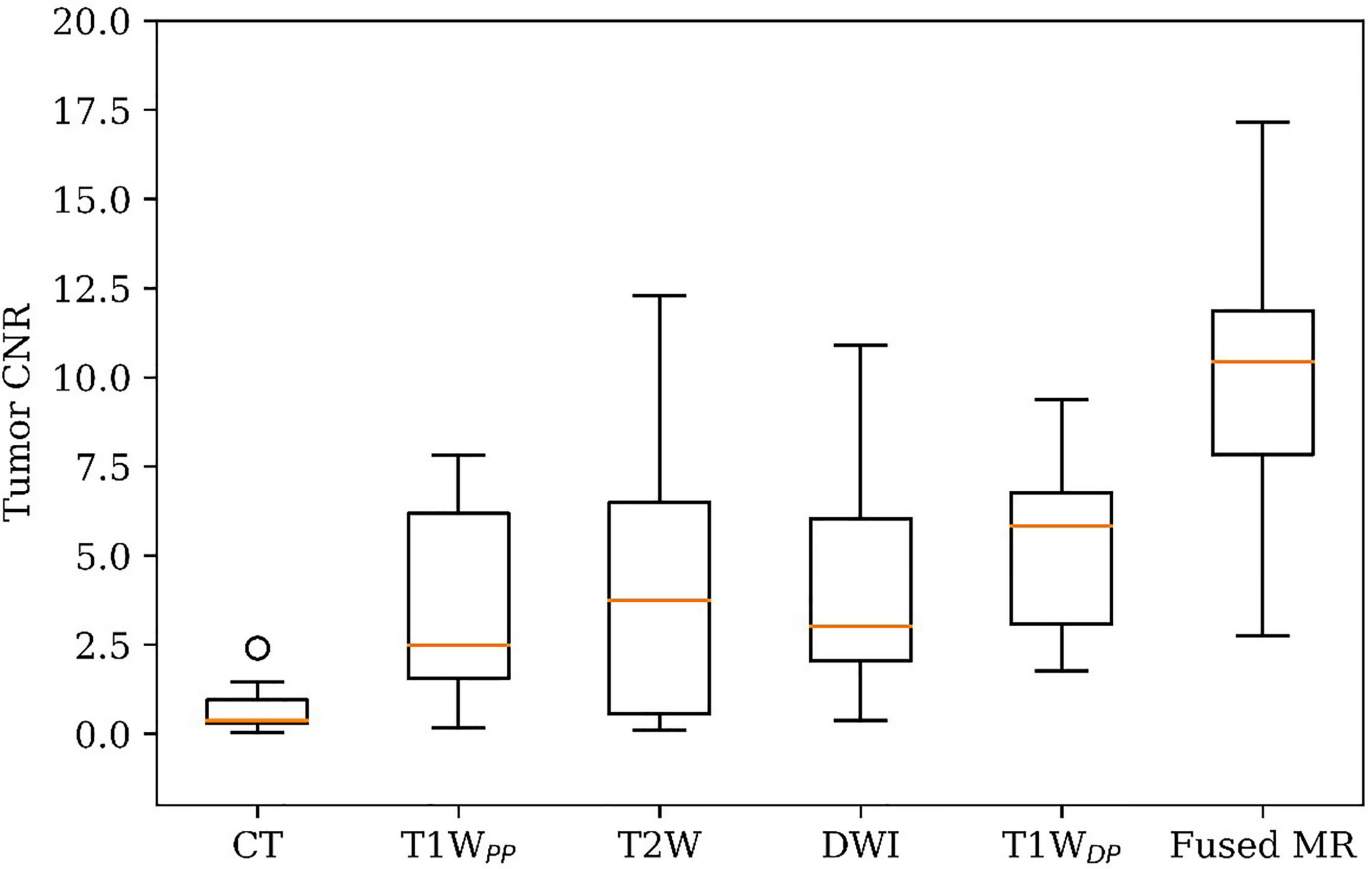
Patient tumor CNR in planning CT, four original MR image sets, and fused MRI. CNR, contrast-to-noise ratio.

**Figure 1** shows the comparison of tumor CNR between the planning CT, the four original MR image sets, and the fused MR images. Firstly, it can be observed that the fused MR images achieved the highest mean tumor CNR (9.82 ± 4.19) among all image sets, leading to a statistically significant enhancement as compared to that of CT (9.82 ± 4.19 vs. 0.72 ± 0.73, p < 0.0005), T1W_PP_ (9.82 ± 4.19 vs. 3.66 ± 2.96, p < 0.005), T2W (9.82 ± 4.19 *vs.* 4.13 ± 3.98, p < 0.001), DWI (9.82 ± 4.19 vs. 4.10 ± 3.17, p < 0.005), and T1W_DP_ (9.82 ± 4.19 vs. 5.25 ± 2.44, p < 0.01) images. Secondly, the inter-patient CV of tumor CNR was the lowest in the fused MR images (42.7%), followed by T1W_DP_ (46.5%), DWI (77.5%), T1W_DP_ (81.0%), T2W (96.2%), and planning CT (101.0%), suggesting that the fused MR images achieved minimum tumor CNR variability between patients. Thirdly, the planning CT images had lower mean tumor CNR than T1W_PP_ (p < 0.01), T2W (p < 0.02), DWI (p < 0.005), and T1W_DP_ (p < 0.0005).

**Figure 2:**
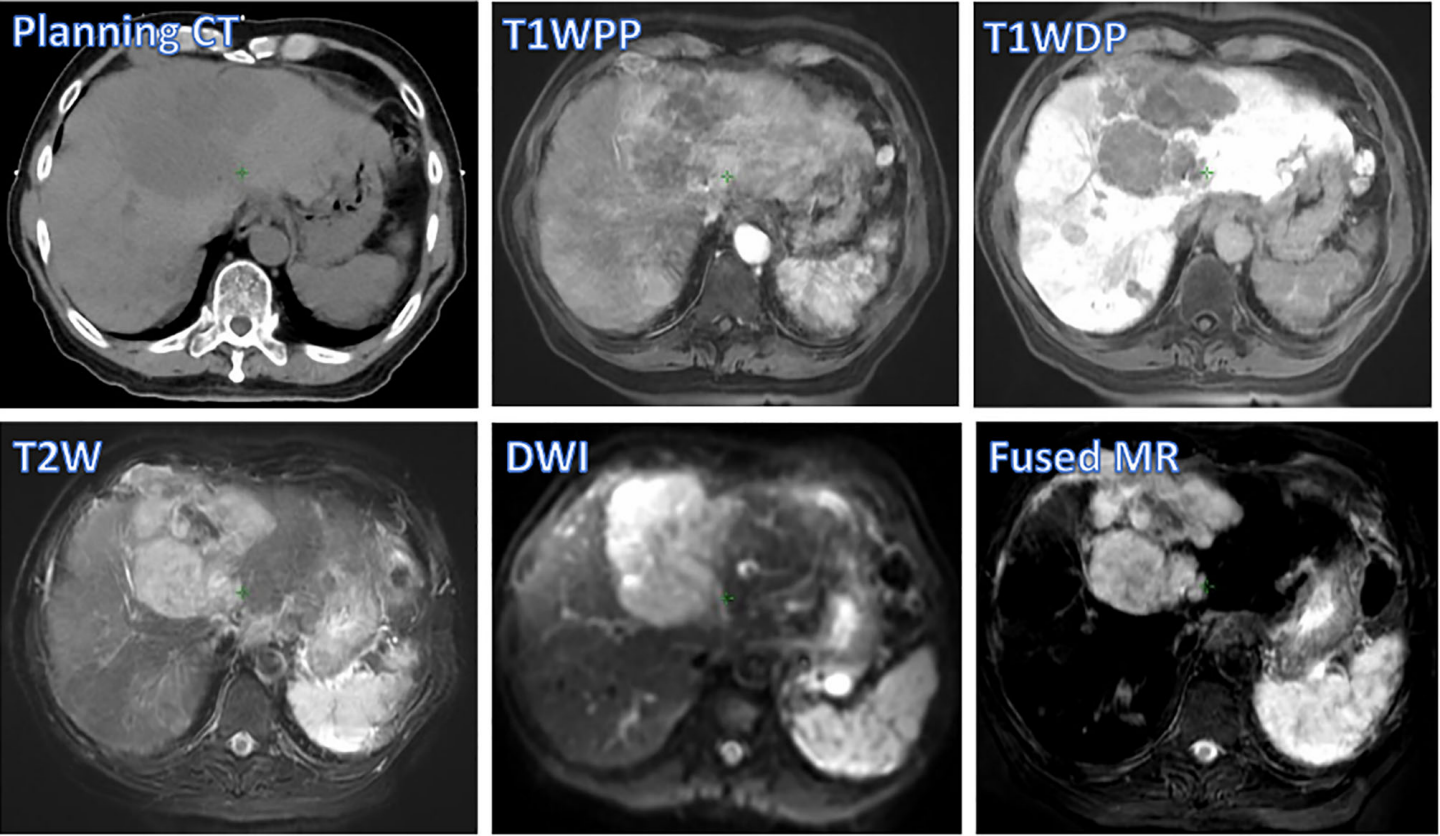
Tumor visibility of a representative patient on various images: planning CT, four original MR sets (T1W_PP_, T1W_DP_, T2W, and DWI), and fused MRI. T1W_PP_, T1-weighted MRI at portal-venous phase; T1W_DP_, T1-weighted MRI at 19-min delayed phase; T2W, T2-weighted; DWI, diffusion-weighted MRI.

**Figure 2** shows different degrees of tumor visibility on the central tumor plane of the planning CT, the four original MR sets (T1W_DP_, T1W_DP_, T2W, DWI), and the fused MR image of a representative patient. For CT and T1W_PP_ images, the tumor and adjacent normal tissue are not clearly discernible. T1W_DP_, T2W, and DWI images show improved tumor contrast. Of note, the fused MR images demonstrated the highest tumor contrast. These findings are in line with the results of quantitative comparisons in terms of CNR (**Figure 1**).

### 3.3 Inter-Observer and Inter-Patient Consistencies of Gross Tumor Volume Delineation

**Figure 3:**
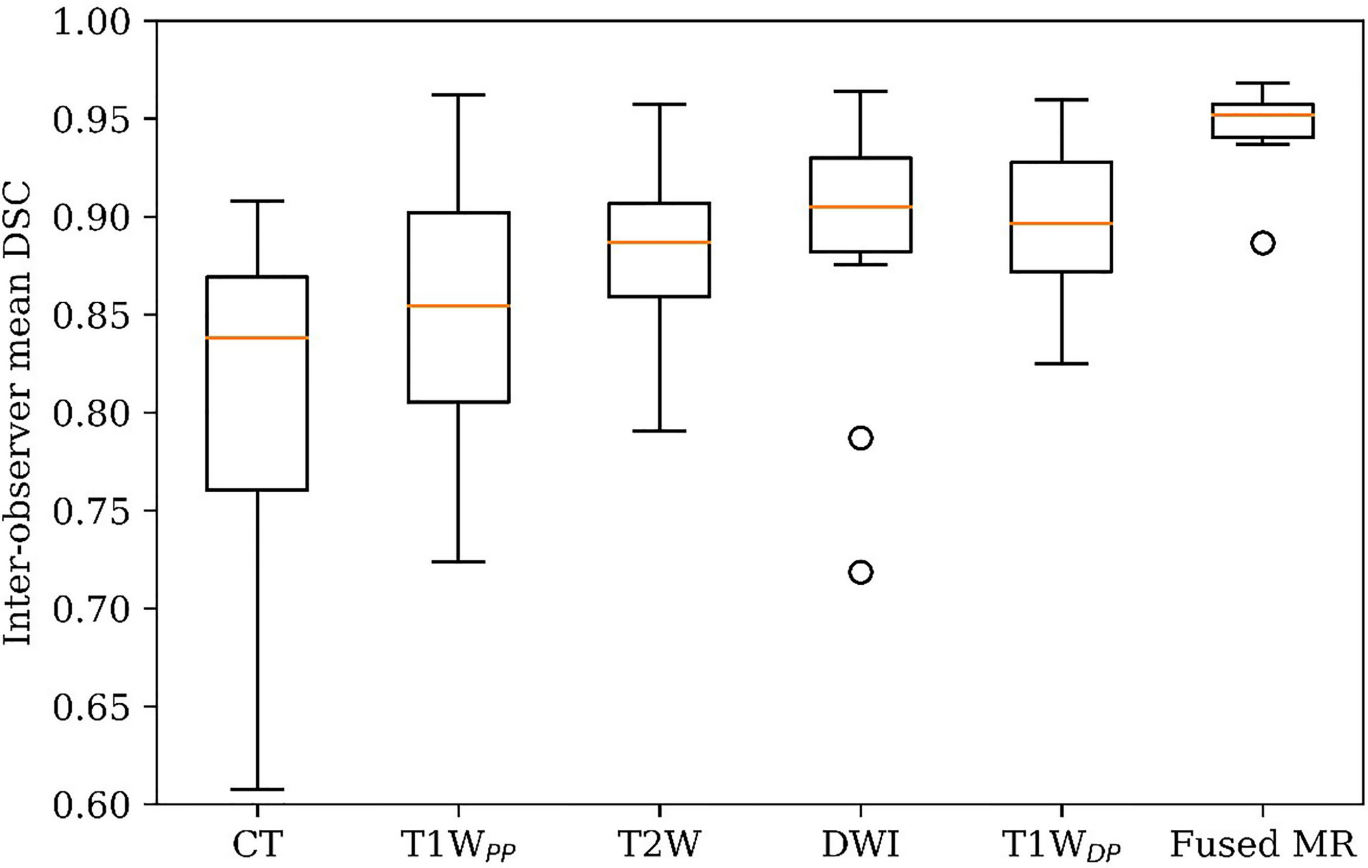
GTV delineation inter-observer mean DSC on planning CT, four original MR image sets, and fused MRI. GTV, gross tumor volume; DSC, Dice similarity coefficient.

**Figure 3** demonstrates the inter-observer mean of the GTV delineation DSC values (DSC_IOmean_) of all ten patients in each of the six studied image sets. Firstly, the fused MR images yielded the highest DSC_IOmean_ (0.95 ± 0.02) among all image sets, leading to a statistically significant enhancement as compared to CT (0.95 ± 0.02 vs. 0.81 ± 0.09, p < 0.0005), T1W_PP_ (0.95 ± 0.02 vs. 0.85 ± 0.08, p < 0.002), T2W (0.95 ± 0.02 vs. 0.88 ± 0.04, p < 0.001), DWI (0.95 ± 0.02 vs. 0.89 ± 0.08, p < 0.05), and T1W_DP_ (0.95 ± 0.02 vs. 0.90 ± 0.04, p < 0.005). Secondly, the inter-patient CV of the DSC_IOmean_ was the lowest in the fused MR images (2.4%), followed by T1W_DP_ (4.6%), T2W (5.1%), DWI (8.5%), T1W_PP_ (9.3%), and planning CT (11.8%). Thirdly, the planning CT images had lower inter-patient mean DSC_IOmean_ of GTV delineations than T2W (p < 0.05), DWI (p < 0.05), and T1W_DP_ (p < 0.05).

**Figure 4:**
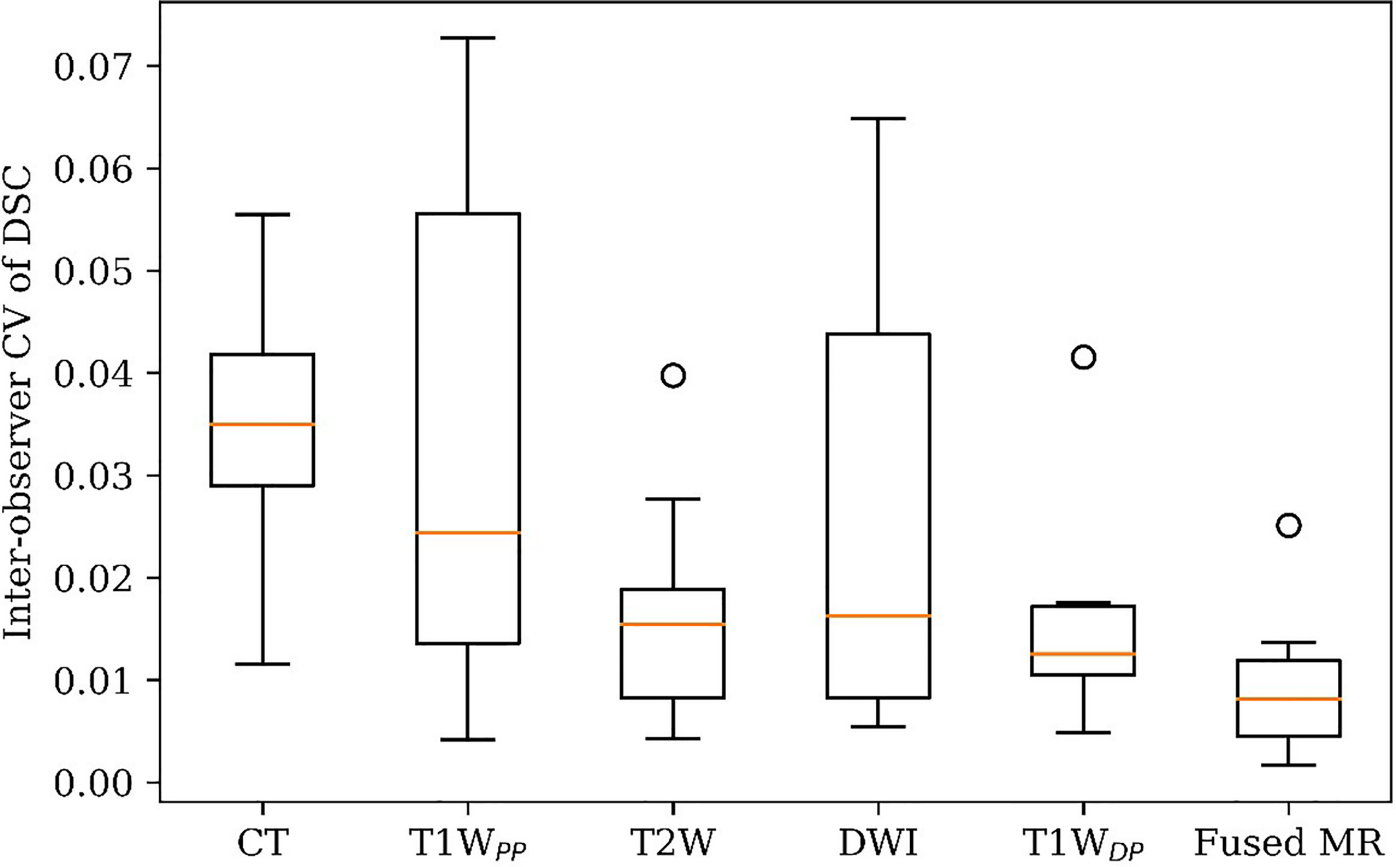
GTV delineation inter-observer coefficient of variation (CV) of DSC on planning CT, four original MR sets, and fused MRI. GTV, gross tumor volume; DSC, Dice similarity coefficient.

**Figure 4** illustrates the inter-observer CV of the GTV delineation DSC values (DSC_IOCV_) of all patients in each of the six image sets. The inter-patient mean DSC_IOCV_ was 3.3%, 3.2%, 1.7%, 2.6%, 1.5%, and 0.9% for planning CT, T1W_PP_, T2W, DWI, T1W_DP_, and fused MR images, respectively. The fused MR exhibited the lowest inter-observer variability in liver HCC tumor delineation in the study.

**Figure 5:**
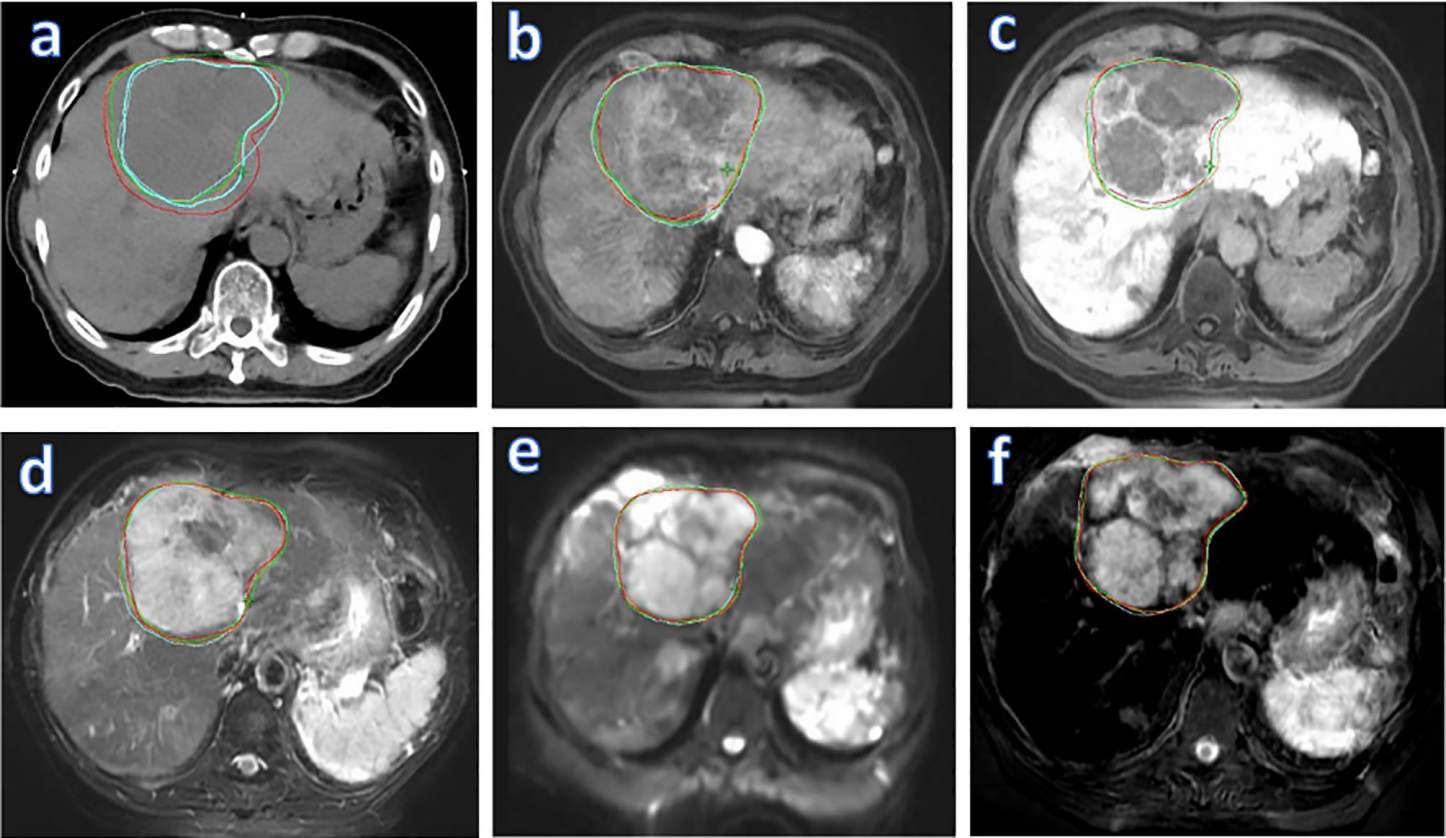
Liver tumor delineation in a representative patient, illustrating different degrees of GTV contouring inter-observer consistency on various images: planning CT **(A)**, four original MR sets (T1W_PP_ **(B)**, T1W_DP_ **(C)**, T2W **(D)**, and DWI **(E)**], and fused MRI **(F)**. GTV, gross tumor volume; T1W_PP_, T1-weighted MRI at portal-venous phase; T1W_DP_, T1-weighted MRI at 19-min delayed phase; T2W, T2-weighted; DWI, diffusion-weighted MRI.

**Figure 5** visualizes GTV contours delineated by all four observers on the (A) planning CT, (B) T1W_PP_, (C) T1W_DP_, (D) T2W, (E) DWI, and (F) fused MR images of a representative patient. The planning CT image (**Figure 5A**) showed the lowest inter-observer consistency in GTV delineations. The four input MR images (**Figures 5B–E**) showed an improved inter-observer consistency in GTV delineation. Notably, the fused MR image (**Figure 5F**) yielded the highest inter-observer consistency in GTV delineation. This agrees with the DSC_IOmean_ and DSC_IOCV_ findings in **Figures 3** and **4**, demonstrating the highest consistency of GTV delineation between observers on the fused MR images.

## 4 Discussion

With the rapid development in imaging and radiation treatment techniques, modern radiotherapy can deliver high ablative radiation dose more accurately to the tumor, leading to improvement of the prognosis for unresectable HCC patients (24–27). Regardless of the chosen radiotherapy technique (intensity-modulated radiotherapy, stereotactic body radiotherapy, etc.), precise tumor delineation is a must and prerequisite for successful radiotherapy treatment. Inaccurate tumor delineation is a major source of errors and can lead to missing of the target during the radiotherapy delivery. It has a significant impact on the dose to the tumor and surrounding normal tissues. Visualization of the tumor and the tumor boundaries within normal tissues is critical for tumor delineation. For HCC, MRI provides superior soft-tissue contrast and therefore more clear tumor boundaries than CT and is a preferred modality for target delineation. MRI has been widely used for image registration with radiotherapy planning CT for tumor delineation in radiotherapy (28). It is an essential clinical procedure in the detection and characterization of HCC, with estimated sensitivity and specificity of 97.4% and 100% (29, 30).

However, there are some limitations of the current practice of MRI-based target delineation in radiotherapy: 1) only one set of MR sequences with a single weighting contrast can be reviewed at a time, making it time-consuming to review multiple sets of MR images during target delineation; 2) tumor contrast may vary significantly between patients and increase the variation and therefore uncertainty in target delineation. The MAMF method, as shown in this study, can be used to enhance MRI tumor contrast as well as its consistency between patients. The MAMF method is therefore a promising tool to overcome the abovementioned obstacles of MRI-based target delineation of HCC. To our best knowledge, this is the first study of systematic evaluation of the clinical efficacy of the MAMF method in HCC. It is worth noting that other challenges of MRI-based radiotherapy also exist, such as the potential of geometry distortion and the lack of electron density information for dose calculation. These areas have been actively studied in the research community (31).

With the MAMF method applied in this study, the fused MRI demonstrated the highest tumor CNR and minimum inter-observer variability. It implies that the detectability and accuracy of tumor delineation of HCC could be enhanced in fused MRI. This improvement could reduce the probability of inaccurate GTV delineation and could affect the clinical outcomes of patients such as tumor local control rate and survival rate.

The four original MR image sets (T1W_PP_, T1W_DP_, T2W, and DWI) were used as input for the MAMF method in this study. These images are commonly used in HCC radiotherapy treatment planning and are typically included in routine abdomen MR imaging protocol. Most HCC lesions can be accurately diagnosed by T1W and T2W MR images (32), and DWI and contrast-enhanced MRI have been shown to be useful contributors to improve the accuracy of liver HCC diagnosis (33–36). It is also worth noting that the proposed MAMF method does not require all four types of MR images as input for performing image fusion. When fewer image modalities are applied as input, the model can be re-trained for generating the fused MR images. Apart from this, the MAMF method is not limited to the four studied MR sequences. Other types of MR images, such as T2/T1-weighted MRI using MR steady-state free precession sequences (22, 37), can also be used as input for MAMF and provide unique contributions to the contrast spectrum of the resulting fused MR images. The clinical efficacy of different MRI sequences combinations for MAMF is yet to be investigated. Besides, the fusion algorithm in the MAMF method is not restricted to specific treatment sites. Further exploration of the generalizability of the MAMF method to other treatment sites is warranted.

On the other hand, it is worth noting that respiratory motion has been demonstrated to adversely influence the quality of thoracic and abdominal images and cause uncertainties in tumor delineation (38, 39). Tremendous efforts have been made to assess a patient’s respiratory motion during radiotherapy and to mitigate its impact on accurate treatment delivery (40–43). Therefore, to minimize the impact of the respiratory motion on the image quality, the acquisitions of the planning CT, T1W_PP_, T1W_DP_, and T2W MR images were performed during breath-holding. DWI images were acquired during the EOE phase using respiratory navigation due to its longer acquisition time. Intrinsically, the acquired “stationary” MR images might still have slight variations in the anatomic position due to potential different breathing depths (44, 45). To tackle this, the four input MR image sets were registered to planning CT by DIR prior to image fusion. It is worth noting that being a group of state-of-the-art registration methods, the DIR methods and their accuracy have been actively studied (46, 47). Advances in the DIR methods would further improve the accuracy of the multisource MRI fusion.

Recently, four-dimensional MRI (4D-MRI) has been an emerging technique for studying the impact of respiratory motion (23, 48, 49). Initial incorporation of 4D-MRI with the MAMF fusion method has been reported (22), suggesting that the MAMF method could be combined with 4D-MRI for enhanced tumor contrast and inter-observer target delineation consistency. One limitation of the study is a relatively small cohort size. As a feasibility study and initial evaluation, the results have demonstrated the capability of tumor CNR enhancements and GTV delineation consistency improvement by the MAMF method. The proposed method can benefit from more validation and testing in a larger cohort study before its consideration for clinical implementation. In future studies, we plan to use more patient cases and digital human phantoms, such as the 4D Digital Extended Cardiac-Torso (XCAT) phantom (50, 51), to more comprehensively evaluate the robustness and accuracy of the proposed method for mobile tumors.

## 5 Conclusion

The preliminary results in ten HCC patients demonstrated that the fused MRI generated using the MAMF method can enhance tumor CNR in HCC as compared with planning CT and four commonly used MR image sets (T1W_PP_, T1W_DP_, T2W, and DWI). The fused MRI can also improve the inter-observer consistency of GTV delineation. The MAMF method holds great promises for HCC tumor delineation and radiotherapy treatment planning.

## Data Availability Statement

The original contributions presented in the study are included in the article/supplementary material. Further inquiries can be directed to the corresponding author.

## Ethics Statement

The studies involving human participants were reviewed and approved by The University of Hong Kong/Hospital Authority Institutional Review Board (HKU/HA HKW IRB). IRB/REC No. UW 21-397. Written informed consent for participation was not required for this study in accordance with the national legislation and the institutional requirements.

## Author Contributions

JC designed and directed the study. JC and ALC applied for study ethical approval. ALC and LZ designed and developed the methods and performed the experiments. ALC, AHC, CYL, AL, and VL managed the patient selection, data collection, and target delineation. ALC, LZ, and CYL performed the data analysis. ALC wrote the first draft. LZ, CL, TL, SKL, and JC revised the manuscript. JC approved the final version. All authors listed have made a substantial, direct, and intellectual contribution to the work and approved it for publication.

## Funding

This work was partly supported by research grants of General Research Fund (GRF 15102118, GRF 15102219), the University Grants Committee; and Health and Medical Research Fund (HMRF 06173276), the Food and Health Bureau, The Government of the Hong Kong Special Administrative Regions.

## Conflict of Interest

The authors declare that the research was conducted in the absence of any commercial or financial relationships that could be construed as a potential conflict of interest.

The reviewer JW declared a shared affiliation, with no collaboration, with two of the authors, LZ, JC, to the handling editor at the time of the review.

## Publisher’s Note

All claims expressed in this article are solely those of the authors and do not necessarily represent those of their affiliated organizations, or those of the publisher, the editors and the reviewers. Any product that may be evaluated in this article, or claim that may be made by its manufacturer, is not guaranteed or endorsed by the publisher.
